# Predictors of Variation in the Cognitive Function Trajectories among Older Adults Living Alone: A Growth Mixture Modeling Approach

**DOI:** 10.3390/healthcare11202750

**Published:** 2023-10-17

**Authors:** Soyoung Park, Seoyoon Lee, Kyu-Hyoung Jeong

**Affiliations:** 1 Department of Social Welfare, Semyung University, 65 Semyung-ro, Jecheon 27136, Republic of Korea; nillyria@semyung.ac.kr (S.P.); jqbrother@semyung.ac.kr (K.-H.J.); 2 Interdisciplinary Graduate Program in Social Welfare Policy, Yonsei University, 50 Yonsei-ro, Seoul 03722, Republic of Korea

**Keywords:** cognitive function, depression, older adults, Korea

## Abstract

Background: Considering the global aging population, this study investigates changes in cognitive function and predictive factors among older adults living alone. Methods: Using data collected from the Korean Longitudinal Study of Aging (KLoSA), the study examines 1217 participants to identify distinct cognitive change patterns and the variables affecting them. Results: Two primary cognitive function change types emerged: “High-Level Declining Type” and “Low-Level Stable Type.” Although the former initially displayed normal cognitive function, it gradually declined over a period of 14 years until it reached mild cognitive impairment (MCI) levels by the year 2020. While the latter group had lower cognitive function from the beginning and remained stable throughout the study. Older age, female gender, rural residence, lower education, lower income, unemployment, and higher levels of depression were linked to a higher likelihood of belonging to the “Low-Level Stable Type”. Conclusions: The findings of these studies emphasize the need for proactive interventions and regular cognitive assessments for older individuals living alone, as cognitive impairment can develop even in individuals whose cognitive abilities are initially good. Also, tailored interventions should target specific demographic and socioeconomic groups to mitigate cognitive decline effectively.

## 1. Introduction

With a growing global population aging, the duration of old age and older stages of life have been extended. With the onset of older age, the quality of life for older adults is becoming increasingly important. Among the factors that diminish the quality of life for older adults is cognitive impairment [1]. Cognitive impairment among older adults not only grants challenges in daily life but also contributes to the rising prevalence of dementia worldwide [2]. In addition to the significant burden imposed on the affected individuals and their caregivers, dementia imposes a significant financial burden on society as a whole [3]. Globally, dementia-related costs were estimated to be USD 1313 billion in 2019, with approximately USD 23,796 in social costs per person living with dementia [4].

A particular concern is the rapid aging of the South Korean population, where there are insufficient preparations for the growing aging population. In 2023, the aging population in South Korea numbered 9,492,582, constituting 18.5% of the total population [5]. Furthermore, the prevalence of dementia among older adults in 2022 exceeded 10.3%, indicating a remarkably high rate of occurrence [6]. It has been shown that dementia not only adversely affects the quality of life of older individuals, but also forces family members to leave their jobs to care for them, resulting in strain on relationships and financial hardship [6]. A significant portion of the societal costs associated with dementia is borne by South Korea, which operates systems to support dementia patients and their families, such as in-home care services and admission to nursing homes and care facilities [7]. Additionally, in order to reduce dementia incidence and alleviate the burden on individuals, families, and society as a whole, social awareness and attention are essential to preventing the rapid decline in cognitive function among older adults.

In particular, older individuals, particularly those living alone, are more likely to suffer from cognitive decline and dementia as a result of a lack of social interaction and shared daily activities [1]. Often, older individuals who live alone find hardship to recognize their own cognitive impairments, making it difficult for them to receive adequate care from family or society, potentially accelerating cognitive decline [8,9]. Furthermore, these individuals, who are less aware of their cognitive impairment and live alone, are more likely to suffer an accident or injury [10,11].

The number of older individuals living alone with cognitive impairment or dementia is expected to increase as the global population of the aging population living alone continues to grow [12,13]. Indeed, the proportion of single-person households in South Korea is growing, with 19.8% of older persons showing such household type [14]. In light of these circumstances, it is imperative that societal attention is drawn to the issue of maintaining cognitive function and preventing cognitive decline in older individuals living alone. A person who lives alone is objectively socially isolated due to the lack of social connections, causing a greater sense of loneliness subjectively [15,16]. There has been an increase in research on the cognitive functions of older adults as a result of the aging of the population, resulting in a heightened awareness of societal concerns and burdens. As part of a study on cognitive function, the following factors were identified as factors affecting cognitive function: gender [17,18,19,20,21], age [22,23], education [22,24,25,26], residential area [27,28,29,30], economic status such as income and occupation [23,26,27,31], physical health [32], and mental health, including depression [22,33,34,35,36,37].

According to recent longitudinal studies, it has been demonstrated that cognitive function changes as individuals age. Cognitive function tends to decline with advancing age [23,38]. In contrast to the modest range of cognitive change associated with increasing age before the age of 70, the rate increased between the ages of 70 and 80 [39]. Furthermore, individuals with initially high cognitive function experienced a slower rate of cognitive decline as they aged. Depression was associated with a faster rate of cognitive decline [40].

It is important to note, however, that not all older people exhibit the same change types in cognitive function. Research has shown that cognitive function changes vary between individuals and that there are several types of changes [41,42,43]. According to Hayden et al. [44], of the study participants aged 75 and older, 65% had little decline in cognitive function over 15 years, 27% had a slight decline, and only 8% had a rapid decline, by insisting on diversity and individual differences. The fact that studies have been conducted on the types of cognitive changes that occur among Korean older individuals is highly encouraging [45,46,47]. However, the number of long-term longitudinal studies targeting all older adults living alone, who are vulnerable in terms of cognitive function, was insufficient among previous studies.

Therefore, the purpose of the study is to explore the types of cognitive changes that occur in cognitively vulnerable older individuals living alone, with the assumption that cognitive function levels and patterns differ from person to person. This study is conducted to categorize older individuals living alone with similar cognitive change trajectories, to examine the characteristics of each type, and to examine factors that cognitive change trajectories. The primary goal of this research is to understand the cognitive change types and predicting factors among older individuals living alone and to discuss social interventions tailored to each type for maintaining cognitive function or preventing dementia. The significance of this study lies in its attempt to categorize the cognitive trajectories of older individuals, particularly those living alone who are vulnerable to the decline in cognitive functions, which has been relatively unexplored in previous research.

## 2. Materials and Methods

### 2.1. Data

Using data collected from the first to eighth waves of the Korean Longitudinal Study of Aging (KLoSA) from 2006 to 2020, this study examined cognitive change types and predicting factors among older individuals living alone. KLoSA is a representative panel survey of the aging population in South Korea. Using various aspects of older adults’ lives, including their social, economic, psychological, demographic, and health-related characteristics, it is designed to provide fundamental data for the development of effective social and economic policies. The subjects of this study are older adults 65 and older who lived alone during the first year of the study, and those who continued to live alone without remarriage or cohabitation throughout the entire study period. In the final analysis, 1217 individuals met the criteria of having cognitive function data available for at least three consecutive years from 2006 (1st wave) to 2020 (8th wave) with no missing values.

### 2.2. Variables

#### 2.2.1. Independent Variable (2006)

This study categorized independent variables into demographic, economical characteristics, and health conditions. First, demographic characteristics included gender (male = 0, female = 1), age (continuous variable), residential area (urban = 0, rural = 1), and education level (elementary school graduation or below = 0, middle school graduation or above = 1). The economic characteristics included household annual income (continuous variable) and employment status (unemployed = 0, employed = 1). The annual household income was log-transformed for a normal distribution. Health characteristics comprise the presence of chronic illnesses (absent = 0, present = 1) and depression, which was measured using the CES-D10 (The Center for Epidemiological Studies-Depression Scale). CES-D is a scale developed by Radloff (1977) [48], and the Korean version CES-D-10, consisting of 10 shortened items, was employed in the Korean Longitudinal Study of Aging to assess depression. The reliability of the depression scale in this study, as measured by Cronbach’s α, was found to be 0.854.

#### 2.2.2. Dependent Variable (2006–2020)

The dependent variable in this study is cognitive function as measured by the Korean Mini-Mental State Examination (K-MMSE). The K-MMSE is an adaptation of the MMSE (Mini-Mental State Examination) originally developed by Folstein, Folstein, and McHugh in 1975 [49], and further adapted by Kang, Na, and Hahn for use in Korea in 1997 [50]. MMSE is a 30-point questionnaire widely used to assess cognitive function in clinical and research settings [51]. A higher score on the K-MMSE indicates a higher level of cognitive functions.

### 2.3. Statistical Analysis

SPSS 28.0 and M-plus 8.0 software programs were used to analyze the data for this study. The following methods and procedures were used in the analysis: Firstly, in order to understand the characteristics of the key variables in the study, descriptive statistics were used. Secondly, to estimate cognitive change among older individuals living alone, a latent growth model (LGM) was utilized, assuming a single group. Model fit was assessed considering model simplicity and insensitivity to sample size. The Tucker–Lewis index (TLI), comparative fit index (CFI), and root mean square error of approximation (RMSEA) were used to evaluate model fit. Thirdly, an analysis of growth mixture models (GMMs) was conducted in order to distinguish between different types of cognitive change among older individuals living alone. The optimal number of cognitive change types was determined through *p*-values of criteria such as Akiakie’s information criteria (AIC), Bayesian information criteria (BIC), sample-size-adjusted BIC (SSABIC), entropy, and the bootstrapped likelihood ratio test (BLRT). Lastly, a logistic regression analysis was conducted to identify predictive factors on cognitive change among older adults living alone.

## 3. Results

### 3.1. Descriptive Statistics

The demographic, economical characteristics, and health conditions of the study participants are presented in Table 1. Among the study participants, 42.8% were male (n = 521), while 57.2% were female (n = 696), indicating that there were more females than males. The average age of the participants was 73.32 years (standard deviation (SD) = 5.69). Among the participants, 74.4% (n = 906) had an education level of elementary school graduation or below, whereas 25.6% (n = 311) had at least a middle school graduation, representing approximately a three-fold difference in numbers. For economic characteristics, the average household annual income was USD 5170.45 (SD = USD 10,286.66). The majority of the participants, 80.9% (n = 984), were unemployed, while 19.1% (n = 233) were employed. As per health conditions, approximately 32.6% (n = 397) of the participants reported having no chronic illnesses, while 67.4% (n = 820) did have chronic illnesses, showing that more than half of the participants had chronic conditions. The average depression score was 1.86 (SD = 0.60).

In a descriptive statistical analysis of cognitive function, it was found that the average cognitive score among older individuals living alone decreased over time (Table 2). Specifically, in 2006, the average score was 22.30 points (SD = 6.06), while in 2020, it had gradually decreased to 19.24 points (SD = 6.54).

#### 3.1.1. Changes in Overall Cognitive Function in Older Adults Living Alone

Prior to conducting the growth mixture model, latent growth modeling was conducted to assess the overall change in cognitive function among older individuals living alone. For this purpose, the no-growth model, linear change model, and quadratic change model were analyzed, and their model fit was compared (Table 3). According to the analysis results, neither the linear change model nor the no-change model fits the data well. However, the quadratic change model demonstrated a good fit with χ^2^ = 79.102 (*p* < 0.001), CFI = 0.982, TLI = 0.982, and RMSEA = 0.040, meeting the fit criteria. As a result, the quadratic change model was adopted.

#### 3.1.2. Trajectories of the Cognitive Function Changes among Older Adults Living Alone

Based on the results of the latent growth model, it was found that the quadratic change model adequately explained the trajectory of cognitive function. Therefore, the quadratic change model was used for estimation in the growth mixture model. The *p*-values from the AIC, BIC, SABIC, entropy, and BLRT were also used to determine the number of latent classes and whether they have at least more than 5% of the cases in a single group when determining the number of cognitive function change types (Table 4).

Based on the results of the analysis, the five-class model had the lowest AIC, BIC, and SSABIC values, and the two-class model had the highest entropy value near 1. In addition, the BLRT was significant for models 2, 3, 4, and 5. The three-, four-, and five-class models, however, had less than 5% of the sample size in one of the classes, suggesting poor fit. Upon considering these fit criteria comprehensively, we concluded that the two-class model would be most appropriate. Therefore, it was selected as the final model for characterizing cognitive function change types in older individuals living alone.

Through the analysis of the growth mixture model, the number of latent classes was determined. The cognitive functions of older adults living alone were classified into two latent classes, each of which was named according to the characteristics of the initial cognitive level and the pattern of cognitive change (Table 5).

**Figure 1:**
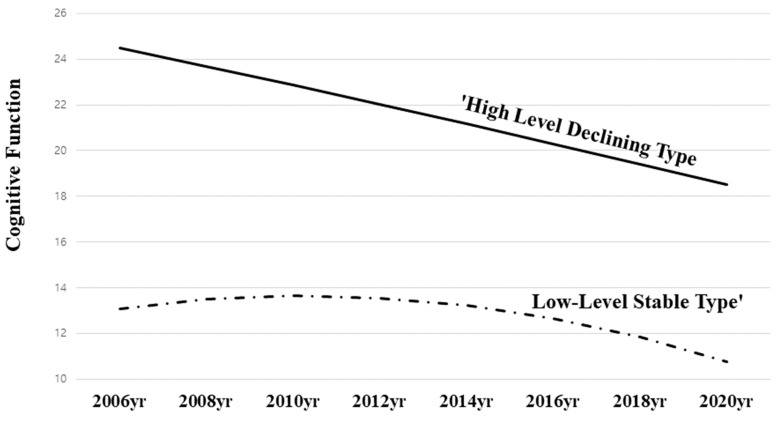
Estimation of changes in cognitive function of the older adults living alone.

Latent Class 1, which comprises 81.6% of the analyzed population (n = 993), showed statistically significant differences in its initial value, 24.473 (*p* < 0.001), and an associated linear rate of change was −0.792 (*p* < 0.001). The quadratic rate of change, however, was not significant. As a result, the cognitive function of these older individuals was higher than the average initially, but over the 14-year period from 2006 to 2020, there was a continual decline in cognitive function. As a result, this class was named ‘High-Level Declining Type’ in light of these characteristics (Figure 1).

In Latent Class 2, which comprises 18.4% of the population (n = 224), there were statistically significant differences in the mean initial value, 13.086 (*p* < 0.001), although the linear and quadratic rates of change were not significant. From 2006 to 2020, the cognitive function of these older individuals remained stable despite their initial cognitive function being lower than the average. Based on these characteristics, this class was referred to as ‘Low-Level Stable Type’.

#### 3.1.3. Determinants of the Types of Cognitive Function Changes among Older Adults Living Alone

A binary logistic regression analysis was conducted to determine the predictive factors of the trajectories in cognitive function among older individuals living alone (Table 6). The analysis results showed that gender (Coef. = 0.815, *p* < 0.001), age (Coef. = 0.123, *p* < 0.001), residential area (Coef. = 0.570, *p* < 0.01), education level (Coef. = −2.115, *p* < 0.001), household income (Coef. = −0.052, *p* < 0.05), employment status (Coef. = −1.067, *p* < 0.01), and depression (Coef. = 0.907, *p* < 0.001) were significant determinants of cognitive function change type. Specifically, being female compared to male, older age, residing in rural areas compared to urban areas, lower education levels, lower household income, not being employed compared to being employed, and higher levels of depression were associated with a higher risk of older individuals living alone belonging to the “Low-Level Stable Type” of cognitive function change type compared to the “High-Level Declining Type”. On the other hand, the presence or absence of chronic illnesses was not a significant determinant of cognitive function change type.

## 4. Discussion

The purpose of this study was to examine the types of cognitive function changes that occur in older adults living alone who are considered cognitively vulnerable, assuming that cognitive levels and patterns of change differ among older adults. In this study, the characteristics of each type of change were examined along with factors predicting these types of changes. According to the results of this study, the average cognitive function of older individuals living alone has gradually declined over time. In contrast to studies on older individuals who have shown no cognitive changes over a 14-year period [44], it aligns with the majority of previous research results that suggest cognitive decline with increasing age [23,38,39]. The results of this study suggest that older individuals living alone require continued social attention and intervention to improve their cognitive function.

The cognitive function change types among older individuals living alone were categorized into two types: “High-Level Declining Type” and “Low-Level Stable Type”. The “High-Level Declining Type”, which accounted for 81.6% of the study participants, had an initial cognitive function mean score exceeding 24 points, indicating no cognitive impairment, but showed a continuous decline in cognitive function over 14 years, reaching slightly above 18 points in the final period. In terms of cognitive function (MMSE), individuals are classified as follows: 24–30 points indicate no cognitive impairment, 18–23 points indicate mild cognitive impairment, and 0–17 points indicate severe cognitive impairment. Initially, the “High-Level Declining Type” displayed normal cognitive function, but as time progressed, the type developed mild cognitive impairment (MCI). Despite the fact that the cognitive function of older adults living alone may be good initially, there is a high probability that mild cognitive impairment (MCI) may develop over time. Therefore, proactive programs aimed at preventing cognitive decline should be implemented for older individuals who have initially good cognitive function. Since older adults living alone may not be able to perceive cognitive decline or impairment due to the absence of family members or friends [11], regular cognitive assessments should be conducted for all older individuals living alone, regardless of their level of cognitive function. It is recommended that appropriate services and programs are connected based on the results of this study in order to maintain cognitive function and prevent impairment.

Those classified as “Low-Level Stable Type” had cognitive function averaged in the 13-point range from the initial assessment, indicating a clear cognitive impairment (SCI) from the beginning. The cognitive impairment persisted for 14 years prior to the last assessment. As a percentage, 18.4% of older adults belong to this type, which is characterized by a clear cognitive impairment from the outset. The prevalence of cognitive impairment among older individuals aged 65 or older is approximately one in five. Therefore, older individuals who have experienced cognitive impairment require increased social attention and alternative forms of social intervention [13], which requires social intervention in a different way from the “High-Level Declining Type”. While cognitive assessment and prevention interventions are necessary for the “High-Level Declining Type”, the “Low-Level Stable Type” already has cognitive impairment from the onset of old age, which requires specialized treatment programs. Older individuals living alone often lack family support, which can exacerbate cognitive impairment [8,9]. To encourage the participation of older individuals, these treatment programs should be community-based and monitored.

Factors predicting cognitive function change types were also identified. As age increases, the probability of belonging to the “Low-Level Stable Type” rather than the “High-Level Declining Type” also increases. This corresponds with previous research indicating that age has a negative impact on cognitive function [22,23]. Therefore, it is imperative to implement interventions to mitigate cognitive decline related to aging, taking into account different age groups among older adults. There should be a classification of older people living alone by age group, interventions should be provided tailored to each age group, and programs for maintaining cognitive function or preventing damage should be implemented especially for those who live alone. According to the results, gender also played a role, and females were more likely to belong “Low-Level Stable Type” than males, which is consistent with most previous research [17,18,19,20]. There is a need for social interventions aimed at female older adults living alone due to the rapid rate of cognitive decline among female [21].

Residents of rural areas were more likely to have a “Low-Level Stable Type” of cognitive function than those living in urban areas. In accordance with previous studies [27,28,29,30], it was found that cognitive function was lower among elderly living in rural areas than in urban areas. Considering that older people living alone in rural areas often fall outside the scope of social services such as social welfare institutions or mental health centers, policy support for implementing cognitive function-related programs aimed at older adults living alone in rural areas is necessary. As a result of the study, the lower the level of education, the greater the probability of belonging to the “Low-Level Stable Type”. Although the result is consistent with conclusions from previous studies [22,25,26] that found that a lower education level accelerated cognitive decline, it is not consistent with Christensen et al. [24] that education has no relation to cognitive function.

The lower the annual income, the higher the probability of belonging to the “Low-Level Stable Type” when not employed, which is consistent with the results of previous studies [26,27] showing that it is easy to maintain cognitive function when income is high and there is employment. An older adult living alone is likely to be economically challenged or unable to work, so policies such as the Elderly Jobs Project are needed to guarantee their income. Depression was also found to be a factors predicting the change in cognitive function of older adults living alone, which is consistent with most previous studies [22,33,34,35,36,37]. According to the results of this study, it is imperative that society provides support for seniors living alone in order to prevent depression, and especially for seniors living alone with cognitive decline, community-based depression-related services should be developed and implemented systematically.

### Limitations

It distinguishes itself from previous studies by examining the cognitive function changes in older individuals living alone over a period of 14 years and exploring the factors that predict them. As a result of the research findings, it is important to present the importance of interventions and intervention strategies in order to maintain cognitive function and delay its decline among older adults living alone. There are, however, some limitations to this study. First of all, this study was unable to control for the external effects of the COVID-19 pandemic during the period from 2006 to 2020 on the cognitive function levels of older individuals living alone. Furthermore, the study was challenged by the attrition of participants over a period of 14 years covered by the secondary data used for analysis. Also, the study relied on secondary data, which limited the variety of independent variables that could be considered. For example, in the case of the “Low-Level Stable Type”, which is one of the types of cognitive function changes derived from the results of this study, it is hard to say that the individual lived without other people or social welfare services or the prolonged absence of assistance for more than 10 years. In the data, it was difficult to find variables that measured support from others or care services, thus these variables could not be used as independent variables. It is recommended to consider a broader range of independent factors in future research.

## 5. Conclusions

This study was conducted on the premise that the level of cognitive function and changes in older adults are different for each individual, as the number of older people increases as a result of population aging and cognitive decline and dementia has emerged as a social problem among older adults. For that purpose, an analysis of the types of change and their predicting factors was conducted. The study identified two types of cognitive change in older people living alone: a “High-Level Declining Type” and a “Low-Level Stable Type”. The “High-Level Declining Type” demonstrated continued declines in cognitive function from a state of no cognitive impairment at the beginning of the investigation to a state of mild cognitive impairment (MCI) by the year 2020. “Low-Level Stable Type” corresponded to cognitive dysfunction (SCI) from the beginning of the investigation and continued to have clear cognitive impairment (SCI) to the end of the survey. As a result of this study, it was possible to verify the types of changes in cognitive function among older individuals living alone who are vulnerable to cognitive decline; there was a need for social intervention in the case of older people living alone, especially those with initially normal cognitive functions that continued to decline rapidly. In addition, gender, age, residential area, education level, annual income, working status, and depression were analyzed as factors predicting the trajectories of cognitive function of older adults living alone. Intervention plans were discussed for each type of change in cognitive function in older adults living alone based on the results of the study.

## Figures and Tables

**Table 1:** Demographic characteristics of study participants (N = 1217).

Variable		Categories	N	%
Demographic characteristics	Gender	Male	521	42.8
		Female	696	57.2
	Age (M(SD))		73.32 (5.69)	
	Residential Area	Urban	765	62.9
		Rural	452	37.1
	Educational Level	Elementary school graduation or below	906	74.4
		Middle school graduation or above	311	25.6
Economical characteristics	USD Annual Household Income (M(SD))		5170.45 (10,286.66)	
	Employment status	Unemployed	984	80.9
		Employed	233	19.1
Health Conditions	Chronic Illness	No	397	32.6
		Yes	820	67.4
	Depression (M(SD))		1.86 (0.60)	

**Table 2:** Descriptive analysis of major variable (N = 1217).

Variable	Min	Max	M	SD
MMSE Score—2006yr	0	30	22.30	6.06
MMSE Score—2008yr	0	30	21.71	6.27
MMSE Score—2010yr	0	30	21.18	6.60
MMSE Score—2012yr	0	30	21.25	6.55
MMSE Score—2014yr	0	30	20.26	6.97
MMSE Score—2016yr	0	30	20.52	6.86
MMSE Score—2018yr	0	30	19.58	7.06
MMSE Score—2020yr	2	30	19.24	6.54

**Table 3:** Goodness of fit of latent growth model of cognitive function.

Model	χ^2^	df	CFI	TLI	RMSEA
No-Growth Model	737.979 ***	34	0.762	0.804	0.130
Linear Change Model	168.367 ***	31	0.954	0.958	0.060
Quadratic Change Model	79.102 ***	27	0.982	0.982	0.040

*** *p* < 0.001.

**Table 4:** Goodness of fit of the growth mixture model.

Class	Model Fit					Groups
	AIC	BIC	SABIC	Entropy	BLRT *p*-Value	N (%)
1	35,045.927	35,132.697	35,078.698	-	-	-
2	34,852.230	34,959.417	34,892.713	0.836	<0.001	993 (81.6), 224 (18.4)
3	34,753.109	34,880.712	34,801.302	0.815	<0.001	967 (79.5), 219 (18.0), 31 (2.5)
4	34,676.223	34,824.243	34,732.127	0.800	<0.001	914 (75.1), 186 (15.3), 94 (7.7), 23 (1.9)
5	34,633.484	34,801.921	34,697.100	0.777	<0.001	786 (64.6), 240 (19.7), 87 (7.1), 82 (6.7), 22 (1.8)

**Table 5:** Estimated initial value and rate of change for each latent class of cognitive function.

Class	N (%)	Parameter Estimates			Change Types
		Initial Value	Linear Rate of Change	Rate of Change in Quadratic Function	
Class 1	993 (81.6)	24.473 ***	−0.792 ***	−0.008	High Level Declining Type
Class 2	224 (18.4)	13.086 ***	0.521	−0.122	Low-Level Stable Type

*** *p* < 0.001.

**Table 6:** Binary logistic regression model of types of change in cognitive function (N = 1217).

Variables		Coef.	S.E.	Exp(B)
Demographic characteristics	Gender (ref. Male)	0.815 ***	0.207	2.259
	Age	0.123 ***	0.015	1.131
	Residential area (ref. Urban)	0.570 **	0.180	1.769
	Educational Level (ref. Elementary school graduation or below)	−2.115 ***	0.438	0.121
Economical characteristics	Annual Household Income (log)	−0.052 *	0.026	0.949
	Employment status (ref. unemployed)	−1.067 **	0.327	0.344
Health Conditions	Chronic illness (ref. No)	−0.185	0.187	0.831
	Depression	0.907 ***	0.138	2.476
constant		−12.431	1.270	0.000

* *p* < 0.05, ** *p* < 0.01, *** *p* < 0.001.

## Data Availability

The datasets generated during and/or analyzed in this study are publicly available upon request from: https://www.koweps.re.kr:442/eng/data/data/list.do (accessed on 12 July 2023).
